# Correction to ‘Resting metabolic rate prediction equations and the validity to assess energy deficiency in the athlete population’

**DOI:** 10.1113/EP094060

**Published:** 2026-06-02

Schofield, K.L., Thorpe, H. & Sims, S.T. (2019). Resting metabolic rate prediction equations and the validity to assess energy deficiency in the athlete population. *Experimental Physiology*, 104(4), 469–475. https://doi.org/10.1113/EP087512

In the originally published article, the equation for males attributed to Mifflin et al. (1990) in Table 1 was incorrectly stated as:

$$\mathrm{RMR}=\left(9.99\times \mathrm{BM}\right)+\left(6.25\times H\right)-\left(5\times A\right).$$

The correct equation is:

$$\mathrm{RMR}=\left(9.99\times \mathrm{BM}\right)+\left(6.25\times H\right)-\left(5\times A\right)+5.$$

We apologise for this error, which has now been corrected.
